# Meetings of Societies

**Published:** 1907-12

**Authors:** 


### MEETINGS OF SOCIETIES.

### Bristol Medico-Chirurgical Society.

*Annual Meeting, October 9th.* 1907.

After a vote of thanks to the retiring President, Mr. James Taylor, had been passed, Dr. Waldo took the chair and gave an introductory address (see p. 289). Later a cordial vote of thanks was given to Dr. Waldo for his able and interesting address.

The Honorary Secretary, Mr. H. F. Mole, read his annual report. The balance in hand, on the ordinary account, was £120 18s. 7d. The Society had lost two members through death, viz. Dr. Markham Skerritt and Mr. H. W. Kendall, and nine members had left the Society owing to removal, &c. Sixteen new members had been elected.

The Editorial Secretary of the *Journal*, Mr. James Taylor, read his annual report.

Mr. Munro Smith read the Library report, which showed that there were 20,989 volumes in the library, and that 237 periodicals were regularly received.

The following officers were chosen for the ensuing year: President-elect—Dr. Michell Clarke; Hon. Secretary—Dr. J. A. Nixon; Members of Committee—Dr. B. M. H. Rogers, Dr. P. Watson Williams, Mr. C. K. Rudge, Dr. James Swain, Dr George Parker, and Prof. Walker Hall; Members of the Library Committee—Mr. C. K. Rudge, Mr. Munro Smith, and Dr. C. F. Carey Coombs.

---

*November 13th,* 1907.

Dr. HENRY WALDO, President, in the Chair.

Nearly the whole time of the meeting was devoted to the consideration of a special report of the Library Committee. Printed copies of this report had been previously posted to the members of the Society. The various suggestions made by the Committee for the improvement of the library were considered seriatim, and except for some minor alterations were approved.

Dr. TEMPLE showed microscopic sections of a **pigmented growth** he had removed two years previously from the prepuce of a boy aged 9. The small tumour had followed an injury. A distinguished pathologist had examined the sections, and pronounced the tumour to be a melanotic sarcoma. Such a growth at the age of nine and in the situation described seemed unique.

J. LACY FIRTH.
J. A. NIXON.

**RECEIPT AND EXPENDITURE ACCOUNT OF THE BRISTOL MEDICO-CHIRURGICAL SOCIETY, SESSION 1906-7.**

| | £ | s. | d. |
|---|---|---|---|
| Carried forward from 1905–6 .. .. .. .. | 147 | 14 | 6 |
| Members' Subscriptions, 1906–7 .. .. .. .. | 157 | 10 | 0 |
| Interest on Deposit Notes | 3 | 4 | 4 |
| Feb. 17.—Excess on Dr. A.'s cheque .. | 0 | 4 | 0 |
| | £308 | 12 | 10 |

| 1906. | £ | s. | d. |
|---|---|---|---|
| Oct. 22.—Grant to Library .. .. .. | 60 | 0 | 0 |
| Stamps .. .. .. | 1 | 0 | 0 |
| Grant to Librarian.. | 5 | 5 | 0 |
| Dec. 19.—Cheque Book | 0 | 2 | 6 |
| Telephone .. .. | 3 | 10 | 0 |
| 1907. | | | |
| Jan. 10.—Grant to Library .. .. .. | 15 | 0 | 0 |
| ,, 18.—Lamp .. .. | 0 | 16 | 3 |
| Feb. 6.—Stamps .. .. | 1 | 0 | 0 |
| May 23.—To Porter .. | 1 | 5 | 3 |
| ,, 24.—For use of lantern .. .. .. | 0 | 15 | 6 |
| Sept. 12.—For use of rooms .. .. .. | 52 | 10 | 0 |
| ,, 17.—Printing .. | 1 | 13 | 6 |
| ,, Do. .. .. | 3 | 11 | 0 |
| ,, 26.—To *Journal*.. | 40 | 5 | 0 |
| Sundries .. | 1 | 0 | 3 |
| | £187 | 14 | 3 |
| Balance as per deposit note No. 375 .. .. | 120 | 18 | 7 |
| | £308 | 12 | 10 |

